# 5-Chloro-*N*′-cyclo­hexyl­idene-3-methyl-1*H*-indole-2-carbohydrazide

**DOI:** 10.1107/S1600536813016899

**Published:** 2013-06-22

**Authors:** Mehmet Akkurt, Muhammet Zopun, Gültaze Çapan, Orhan Büyükgüngör

**Affiliations:** a Department of Physics, Faculty of Sciences, Erciyes University, 38039 Kayseri, Turkey; b Department of Pharmaceutical Chemistry, Faculty of Pharmacy, Istanbul University, 34116 Beyazit, Istanbul, Turkey; c Department of Physics, Faculty of Arts and Sciences, Ondokuz Mayıs University, 55139 Samsun, Turkey

## Abstract

In the title compound, C_16_H_18_ClN_3_O, the cyclo­hexane ring adopts a distorted chair conformation. In the crystal, pairs of mol­ecules are linked by N—H⋯O hydrogen bonds into inversion dimers, forming *R*_2_^2^(10) ring motifs. These dimers are connected through C—H⋯N hydrogen bonds into chains along the *a* axis, forming layers parallel to (101).

## Related literature

For the design, synthesis and characterization of some bioactive indole derivatives, see: Akkurt *et al.* (2009 ▶, 2010 ▶); Cihan-Üstündağ & Çapan (2012 ▶); Güzel *et al.* (2006 ▶); Kaynak *et al.* (2005 ▶). For puckering analysis, see: Cremer & Pople (1975 ▶). For hydrogen-bond motifs, see: Bernstein *et al.* (1995 ▶).
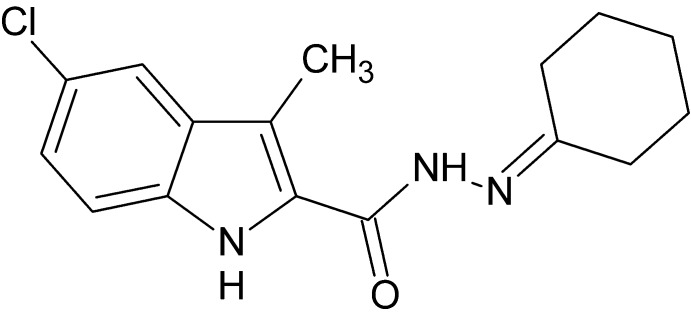


## Experimental

### 

#### Crystal data

C_16_H_18_ClN_3_O
*M* *_r_* = 303.78
Triclinic, 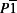

*a* = 5.2727 (5) Å
*b* = 9.7977 (9) Å
*c* = 15.2380 (15) Å
α = 102.229 (7)°
β = 95.732 (8)°
γ = 92.332 (7)°
*V* = 763.94 (13) Å^3^
*Z* = 2
Mo *K*α radiation
μ = 0.25 mm^−1^
*T* = 296 K
0.76 × 0.36 × 0.02 mm

#### Data collection

Stoe IPDS 2 diffractometer
Absorption correction: integration (*X-RED32*; Stoe & Cie, 2002 ▶) *T*_min_ = 0.831, *T*_max_ = 0.995
7177 measured reflections
2929 independent reflections
1684 reflections with *I* > 2σ(*I*)
*R*_int_ = 0.065

#### Refinement

*R*[*F* ^2^ > 2σ(*F* ^2^)] = 0.053
*wR*(*F* ^2^) = 0.129
*S* = 1.01
2929 reflections
195 parameters
H atoms treated by a mixture of independent and constrained refinement
Δρ_max_ = 0.15 e Å^−3^
Δρ_min_ = −0.16 e Å^−3^

### 

Data collection: *X-AREA* (Stoe & Cie, 2002 ▶); cell refinement: *X-AREA*; data reduction: *X-RED32* (Stoe & Cie, 2002 ▶); program(s) used to solve structure: *SHELXS97* (Sheldrick, 2008 ▶); program(s) used to refine structure: *SHELXL97* (Sheldrick, 2008 ▶); molecular graphics: *ORTEP-3 for Windows* (Farrugia, 2012 ▶); software used to prepare material for publication: *WinGX* (Farrugia, 2012 ▶).

## Supplementary Material

Crystal structure: contains datablock(s) global, I. DOI: 10.1107/S1600536813016899/sj5335sup1.cif

Structure factors: contains datablock(s) I. DOI: 10.1107/S1600536813016899/sj5335Isup2.hkl

Supplementary material file. DOI: 10.1107/S1600536813016899/sj5335Isup3.cml

Additional supplementary materials:  crystallographic information; 3D view; checkCIF report

## Figures and Tables

**Table 1:** Hydrogen-bond geometry (Å, °)

*D*—H⋯*A*	*D*—H	H⋯*A*	*D*⋯*A*	*D*—H⋯*A*
N1—H1⋯O1^i^	0.86	2.03	2.826 (3)	153
C12—H12*B*⋯N3^ii^	0.97	2.59	3.476 (4)	152

Symmetry codes: (i) 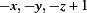; (ii) 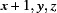.

**Fig. 1.**
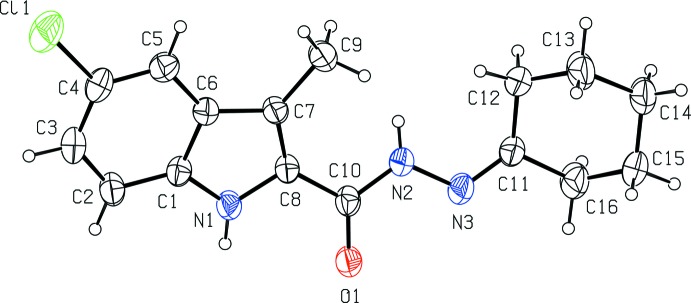
The title molecule shown the atom labelling scheme. Displacement ellipsoids for non-H atoms are drawn at the 30% probability level.

**Fig. 2.**
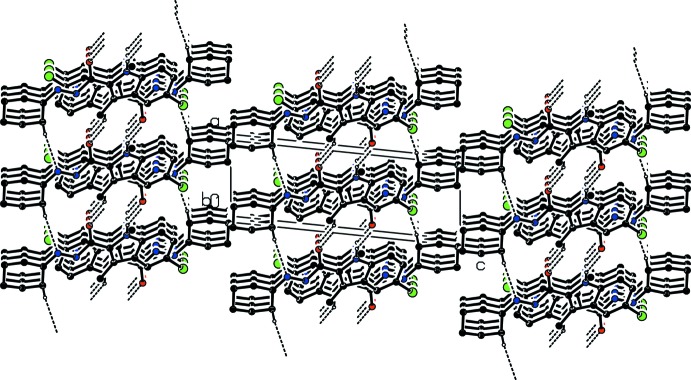
View of the packing of the title compound with the N—H···O dimers, down the b axis. H atoms not participating in hydrogen bonding have been omitted for clarity and hydrogen bonds are drawn as dashed lines.

## supplementary crystallographic information

### Comment

Cyclohexylidenehydrazides are of interest, both as potential intermediates for the synthesis of novel heterocyclic systems and as pharmacologically active agents. We have recently reported on the synthesis, antitituberculosis and anticancer properties of cyclohexylidenehydrazides and spirothiazolidinones with an indole core (Cihan-Üstündağ & Çapan, 2012). As a continuation of our program directed towards the design, synthesis and characterization of bioactive indole derivatives (Akkurt *et al.*, 2009, 2010; Güzel *et al.*, 2006; Kaynak *et al.*, 2005), we report here the synthesis, spectral and analytical data and crystal structure of the title compound.

In the title compound (I), (Fig. 1), the nine-membered 1*H*-indole ring (N1/C1–C8) is essentially planar with maximum deviations of 0.019 (3) Å for C3, 0.017 (3) Å for C7 and -0.017 (3) Å for C1]. The cyclohexane ring (C11–C16) of (I) adopts a distorted chair conformation [the puckering parameters (Cremer & Pople, 1975) are Q_T_ = 0.508 (4) Å, θ = 10.2 (5)° and φ = 193 (2) °]. The C7–C8–C10–N2, C7–C8–C10–O1, N1–C8–C10–O1, N1–C8–C10–N2, C8–C10–N2–N3 and C10–N2–N3–C11 torsion angles are -19.5 (5), 158.3 (3), -14.4 (4), 167.7 (3), -179.6 (2) and -172.8 (3)°, respectively.

In the crystal, pairs of N—H···O hydrogen bonds link molecules into inversion dimers, with the *R*^2^_2_(10) ring motifs (Table 1, Fig. 2; Bernstein *et al.*, 1995). These dimers connect to each other through C—H···N hydrogen bonds as chains along the *a* axis, forming layers parallel to the (101) plane. In the crystal structure, π-π and C—H···π interactions were not observed.

### Experimental

A mixture of 5-chloro-3-methyl-1*H*-indole-2-carbohydrazide (0.005 mol) and cyclohexanone (0.006 mol) in 15 ml of absolute ethanol was heated under reflux for 3 h. The crude product obtained on cooling was filtered and purified by recrystallization from ethanol. [Yield: 91.4%, m.p.: 505–507 K].

### Refinement

H atoms bonded to C atoms were positioned geometrically with C—H = 0.93, 0.96 and 0.97 Å, and refined using a riding model with *U*_iso_(H) = 1.2 or 1.5*U*_eq_(C). The H atom (H1) of the one of the two amide groups was positioned geometrically with N—H = 0.86 Å, and refined using a riding model with *U*_iso_(H) = 1.2*U*_eq_(N). The H atom (H2A) of the other amide group was found in a difference Fourier map, restrained with N—H = 0.82 (3) Å and refined with *U*_iso_ = 1.2*U*_eq_(N).

### Crystal data

**Table:**

C_16_H_18_ClN_3_O	*Z* = 2
*M**_r_* = 303.78	*F*(000) = 320
Triclinic, *P*1	*D*_x_ = 1.321 Mg m^−^^3^
Hall symbol: -P 1	Mo *K*α radiation, λ = 0.71073 Å
*a* = 5.2727 (5) Å	Cell parameters from 9705 reflections
*b* = 9.7977 (9) Å	θ = 2.1–28.0°
*c* = 15.2380 (15) Å	µ = 0.25 mm^−^^1^
α = 102.229 (7)°	*T* = 296 K
β = 95.732 (8)°	Plate, colourless
γ = 92.332 (7)°	0.76 × 0.36 × 0.02 mm
*V* = 763.94 (13) Å^3^	

### Data collection

**Table:**

Stoe IPDS 2 diffractometer	2929 independent reflections
Radiation source: sealed X-ray tube, 12 x 0.4 mm long-fine focus	1684 reflections with *I* > 2σ(*I*)
Plane graphite monochromator	*R*_int_ = 0.065
Detector resolution: 6.67 pixels mm^-1^	θ_max_ = 26.0°, θ_min_ = 2.1°
ω scans	*h* = −6→6
Absorption correction: integration (*X-RED32*; Stoe & Cie, 2002)	*k* = −12→12
*T*_min_ = 0.831, *T*_max_ = 0.995	*l* = −18→18
7177 measured reflections	

### Refinement

**Table:**

Refinement on *F*^2^	Primary atom site location: structure-invariant direct methods
Least-squares matrix: full	Secondary atom site location: difference Fourier map
*R*[*F*^2^ > 2σ(*F*^2^)] = 0.053	Hydrogen site location: inferred from neighbouring sites
*wR*(*F*^2^) = 0.129	H atoms treated by a mixture of independent and constrained refinement
*S* = 1.01	*w* = 1/[σ^2^(*F*_o_^2^) + (0.0554*P*)^2^] where *P* = (*F*_o_^2^ + 2*F*_c_^2^)/3
2929 reflections	(Δ/σ)_max_ < 0.001
195 parameters	Δρ_max_ = 0.15 e Å^−^^3^
0 restraints	Δρ_min_ = −0.16 e Å^−^^3^

### Special details

**Table:**

Experimental. UV (EtOH) λ_max_(nm)(ε) = 210.2 (11298); 230.6 (15341); 303.4 (14665). IR(KBr)ν = 3278 (N—H); 1639 (C=O);1624, 1537, 1514, 1471 (C=N, C=C) cm^-1^. ^1^H-NMR (500 MHz) (DMSO-d_6_ / TMS) d =1.56–1.72 (6*H*, m, CH_2_-cyc.*), 2.32 (2*H*, t, J=6.8 Hz, CH_2_-cyc.) 2.42–2.48 (5*H*, m, CH_2_-cyc. and 3-CH_3_-ind.*), 7.20 (1*H*, dd, J=8.7, 1.9 Hz, H6-ind.), 7.41 (1*H*, d, J=8.7 Hz, H7-ind.), 7.66 (1*H*, d, J=1.9 Hz, H4-ind.), 10.30 (1*H*, s, CONH), 11.50 (1*H*, s, NH-ind.) p.p.m.. MS (ESI–) m/*z* (%) = 302 ([M—H]^-^, 100). Analysis calculated for C_16_H_18_ClN_3_O: C 63.26, H 5.97, N 13.83%. Found: C 63.12, H 5.97, N 13.86%.(*cyc. = cyclohexylidene, ind. = indole).
Geometry. Bond distances, angles *etc*. have been calculated using the rounded fractional coordinates. All su's are estimated from the variances of the (full) variance-covariance matrix. The cell e.s.d.'s are taken into account in the estimation of distances, angles and torsion angles
Refinement. Refinement on *F*^2^ for ALL reflections except those flagged by the user for potential systematic errors. Weighted *R*-factors *wR* and all goodnesses of fit *S* are based on *F*^2^, conventional *R*-factors *R* are based on *F*, with *F* set to zero for negative *F*^2^. The observed criterion of *F*^2^ > σ(*F*^2^) is used only for calculating -*R*-factor-obs *etc*. and is not relevant to the choice of reflections for refinement. *R*-factors based on *F*^2^ are statistically about twice as large as those based on *F*, and *R*-factors based on ALL data will be even larger.

### Fractional atomic coordinates and isotropic or equivalent isotropic displacement parameters (Å^2^)

**Table:**

	*x*	*y*	*z*	*U*_iso_*/*U*_eq_	
Cl1	0.5891 (2)	0.46594 (11)	0.20802 (7)	0.1086 (4)	
O1	0.1519 (4)	0.0786 (2)	0.61618 (12)	0.0717 (7)	
N1	0.2262 (4)	0.1409 (2)	0.45351 (13)	0.0589 (8)	
N2	0.5585 (5)	0.1489 (3)	0.67430 (15)	0.0597 (8)	
N3	0.5305 (4)	0.1037 (2)	0.75354 (15)	0.0628 (8)	
C1	0.2826 (5)	0.2059 (3)	0.38687 (17)	0.0538 (8)	
C2	0.1513 (6)	0.1988 (3)	0.30110 (18)	0.0654 (10)	
C3	0.2502 (6)	0.2795 (3)	0.24870 (19)	0.0705 (11)	
C4	0.4744 (6)	0.3636 (3)	0.27889 (19)	0.0691 (11)	
C5	0.6043 (5)	0.3719 (3)	0.3623 (2)	0.0648 (10)	
C6	0.5060 (4)	0.2920 (3)	0.41855 (17)	0.0508 (8)	
C7	0.5838 (4)	0.2775 (3)	0.50912 (16)	0.0512 (8)	
C8	0.4070 (5)	0.1827 (3)	0.52722 (16)	0.0511 (8)	
C9	0.8061 (5)	0.3606 (3)	0.5689 (2)	0.0686 (10)	
C10	0.3624 (5)	0.1311 (3)	0.60883 (17)	0.0543 (9)	
C11	0.7122 (5)	0.1360 (3)	0.81721 (18)	0.0597 (9)	
C12	0.9530 (6)	0.2231 (4)	0.8204 (2)	0.0866 (13)	
C13	1.0168 (7)	0.3247 (4)	0.9086 (2)	0.0993 (16)	
C14	1.0052 (7)	0.2603 (4)	0.9900 (2)	0.0876 (13)	
C15	0.7463 (6)	0.1875 (4)	0.9862 (2)	0.0837 (13)	
C16	0.6818 (6)	0.0800 (4)	0.9003 (2)	0.0820 (11)	
H1	0.09730	0.08260	0.45020	0.0710*	
H2	0.00300	0.14140	0.28090	0.0790*	
H2A	0.701 (5)	0.174 (3)	0.6650 (17)	0.054 (8)*	
H3	0.16670	0.27840	0.19180	0.0850*	
H5	0.75350	0.42900	0.38110	0.0780*	
H9A	0.85040	0.44140	0.54590	0.1030*	
H9B	0.94970	0.30360	0.56980	0.1030*	
H9C	0.76010	0.39010	0.62910	0.1030*	
H12A	0.93570	0.27410	0.77230	0.1040*	
H12B	1.09240	0.16220	0.80990	0.1040*	
H13A	0.89910	0.39870	0.91220	0.1190*	
H13B	1.18740	0.36690	0.91050	0.1190*	
H14A	1.13590	0.19360	0.99100	0.1050*	
H14B	1.03740	0.33270	1.04480	0.1050*	
H15A	0.74350	0.14270	1.03710	0.1010*	
H15B	0.61810	0.25620	0.99110	0.1010*	
H16A	0.50660	0.04370	0.89750	0.0980*	
H16B	0.79110	0.00290	0.90090	0.0980*	

### Atomic displacement parameters (Å^2^)

**Table:**

	*U*^11^	*U*^22^	*U*^33^	*U*^12^	*U*^13^	*U*^23^
Cl1	0.1362 (8)	0.1232 (8)	0.0844 (7)	−0.0039 (6)	0.0217 (6)	0.0607 (6)
O1	0.0767 (12)	0.0851 (14)	0.0498 (11)	−0.0264 (11)	−0.0034 (9)	0.0179 (10)
N1	0.0662 (13)	0.0625 (14)	0.0445 (12)	−0.0171 (11)	−0.0010 (10)	0.0109 (10)
N2	0.0590 (13)	0.0774 (16)	0.0448 (13)	−0.0043 (12)	0.0028 (11)	0.0210 (11)
N3	0.0722 (14)	0.0709 (15)	0.0491 (13)	−0.0018 (12)	0.0054 (12)	0.0236 (12)
C1	0.0654 (15)	0.0521 (15)	0.0427 (14)	0.0010 (13)	0.0064 (12)	0.0080 (12)
C2	0.0775 (17)	0.0694 (18)	0.0455 (15)	−0.0025 (15)	−0.0039 (14)	0.0102 (13)
C3	0.090 (2)	0.077 (2)	0.0444 (15)	0.0084 (17)	0.0005 (15)	0.0155 (14)
C4	0.0879 (19)	0.0721 (19)	0.0551 (18)	0.0077 (16)	0.0153 (16)	0.0274 (15)
C5	0.0675 (16)	0.0657 (17)	0.0633 (18)	−0.0028 (14)	0.0096 (14)	0.0193 (14)
C6	0.0536 (13)	0.0520 (15)	0.0471 (14)	0.0049 (12)	0.0050 (11)	0.0116 (12)
C7	0.0511 (13)	0.0536 (15)	0.0463 (14)	0.0012 (12)	0.0006 (11)	0.0079 (12)
C8	0.0570 (14)	0.0540 (15)	0.0407 (14)	0.0021 (13)	0.0014 (11)	0.0086 (12)
C9	0.0648 (16)	0.078 (2)	0.0613 (17)	−0.0071 (15)	−0.0059 (14)	0.0197 (15)
C10	0.0659 (16)	0.0502 (15)	0.0431 (14)	−0.0023 (13)	0.0016 (13)	0.0054 (12)
C11	0.0635 (15)	0.0719 (18)	0.0463 (15)	0.0060 (14)	0.0073 (13)	0.0177 (13)
C12	0.0652 (17)	0.143 (3)	0.0556 (18)	−0.0114 (19)	0.0048 (14)	0.0348 (19)
C13	0.112 (3)	0.122 (3)	0.065 (2)	−0.040 (2)	−0.0189 (19)	0.045 (2)
C14	0.099 (2)	0.112 (3)	0.0530 (18)	−0.013 (2)	−0.0066 (17)	0.0313 (18)
C15	0.092 (2)	0.116 (3)	0.0491 (17)	0.000 (2)	0.0086 (16)	0.0325 (18)
C16	0.097 (2)	0.092 (2)	0.0633 (19)	−0.0084 (19)	−0.0014 (17)	0.0386 (18)

### Geometric parameters (Å, º)

**Table:**

Cl1—C4	1.754 (3)	C12—C13	1.492 (5)
O1—C10	1.230 (3)	C13—C14	1.511 (5)
N1—C1	1.358 (3)	C14—C15	1.504 (5)
N1—C8	1.378 (3)	C15—C16	1.495 (5)
N2—N3	1.390 (3)	C2—H2	0.9300
N2—C10	1.342 (4)	C3—H3	0.9300
N3—C11	1.272 (3)	C5—H5	0.9300
N1—H1	0.8600	C9—H9A	0.9600
N2—H2A	0.82 (3)	C9—H9B	0.9600
C1—C2	1.403 (4)	C9—H9C	0.9600
C1—C6	1.402 (4)	C12—H12A	0.9700
C2—C3	1.361 (4)	C12—H12B	0.9700
C3—C4	1.393 (4)	C13—H13A	0.9700
C4—C5	1.367 (4)	C13—H13B	0.9700
C5—C6	1.399 (4)	C14—H14A	0.9700
C6—C7	1.438 (4)	C14—H14B	0.9700
C7—C9	1.504 (4)	C15—H15A	0.9700
C7—C8	1.377 (4)	C15—H15B	0.9700
C8—C10	1.474 (4)	C16—H16A	0.9700
C11—C16	1.503 (4)	C16—H16B	0.9700
C11—C12	1.492 (4)		
			
C1—N1—C8	109.6 (2)	C3—C2—H2	121.00
N3—N2—C10	120.0 (2)	C2—C3—H3	119.00
N2—N3—C11	117.5 (2)	C4—C3—H3	120.00
C8—N1—H1	125.00	C4—C5—H5	121.00
C1—N1—H1	125.00	C6—C5—H5	121.00
N3—N2—H2A	118.7 (18)	C7—C9—H9A	109.00
C10—N2—H2A	120.5 (18)	C7—C9—H9B	109.00
N1—C1—C6	107.7 (2)	C7—C9—H9C	109.00
C2—C1—C6	122.3 (3)	H9A—C9—H9B	109.00
N1—C1—C2	130.0 (3)	H9A—C9—H9C	109.00
C1—C2—C3	117.2 (3)	H9B—C9—H9C	110.00
C2—C3—C4	121.1 (3)	C11—C12—H12A	109.00
Cl1—C4—C3	118.5 (2)	C11—C12—H12B	109.00
Cl1—C4—C5	119.0 (2)	C13—C12—H12A	109.00
C3—C4—C5	122.5 (3)	C13—C12—H12B	109.00
C4—C5—C6	118.0 (3)	H12A—C12—H12B	108.00
C1—C6—C5	119.0 (2)	C12—C13—H13A	109.00
C5—C6—C7	133.4 (2)	C12—C13—H13B	109.00
C1—C6—C7	107.6 (2)	C14—C13—H13A	109.00
C6—C7—C8	105.7 (2)	C14—C13—H13B	109.00
C6—C7—C9	123.9 (2)	H13A—C13—H13B	108.00
C8—C7—C9	130.3 (2)	C13—C14—H14A	110.00
C7—C8—C10	133.6 (2)	C13—C14—H14B	110.00
N1—C8—C7	109.5 (2)	C15—C14—H14A	110.00
N1—C8—C10	116.6 (2)	C15—C14—H14B	110.00
O1—C10—C8	120.5 (2)	H14A—C14—H14B	108.00
N2—C10—C8	116.7 (2)	C14—C15—H15A	109.00
O1—C10—N2	122.8 (3)	C14—C15—H15B	109.00
N3—C11—C12	128.4 (3)	C16—C15—H15A	109.00
N3—C11—C16	116.3 (3)	C16—C15—H15B	109.00
C12—C11—C16	115.3 (2)	H15A—C15—H15B	108.00
C11—C12—C13	112.6 (3)	C11—C16—H16A	109.00
C12—C13—C14	113.9 (3)	C11—C16—H16B	109.00
C13—C14—C15	109.9 (3)	C15—C16—H16A	109.00
C14—C15—C16	111.7 (3)	C15—C16—H16B	109.00
C11—C16—C15	113.3 (3)	H16A—C16—H16B	108.00
C1—C2—H2	121.00		
			
C1—N1—C8—C10	174.9 (2)	C4—C5—C6—C7	−177.9 (3)
C8—N1—C1—C2	−178.3 (3)	C5—C6—C7—C9	3.2 (5)
C8—N1—C1—C6	0.0 (3)	C5—C6—C7—C8	179.7 (3)
C1—N1—C8—C7	0.5 (3)	C1—C6—C7—C8	0.8 (3)
N3—N2—C10—C8	179.6 (2)	C1—C6—C7—C9	−175.7 (2)
N3—N2—C10—O1	1.8 (4)	C6—C7—C8—C10	−173.9 (3)
C10—N2—N3—C11	−172.8 (3)	C9—C7—C8—C10	2.3 (5)
N2—N3—C11—C16	−177.2 (3)	C9—C7—C8—N1	175.4 (3)
N2—N3—C11—C12	2.9 (4)	C6—C7—C8—N1	−0.8 (3)
N1—C1—C6—C7	−0.5 (3)	N1—C8—C10—N2	167.7 (3)
C6—C1—C2—C3	0.2 (4)	C7—C8—C10—O1	158.3 (3)
N1—C1—C6—C5	−179.6 (2)	C7—C8—C10—N2	−19.5 (5)
C2—C1—C6—C7	178.0 (3)	N1—C8—C10—O1	−14.4 (4)
C2—C1—C6—C5	−1.1 (4)	N3—C11—C12—C13	136.1 (3)
N1—C1—C2—C3	178.2 (3)	C16—C11—C12—C13	−43.7 (4)
C1—C2—C3—C4	1.0 (4)	N3—C11—C16—C15	−134.0 (3)
C2—C3—C4—Cl1	−179.5 (2)	C12—C11—C16—C15	45.9 (4)
C2—C3—C4—C5	−1.1 (5)	C11—C12—C13—C14	49.1 (4)
C3—C4—C5—C6	0.1 (4)	C12—C13—C14—C15	−55.6 (4)
Cl1—C4—C5—C6	178.5 (2)	C13—C14—C15—C16	56.2 (4)
C4—C5—C6—C1	1.0 (4)	C14—C15—C16—C11	−52.0 (4)

### Hydrogen-bond geometry (Å, º)

**Table:**

*D*—H···*A*	*D*—H	H···*A*	*D*···*A*	*D*—H···*A*
N1—H1···O1^i^	0.86	2.03	2.826 (3)	153
C12—H12*A*···N2	0.97	2.47	2.842 (4)	102
C12—H12*B*···N3^ii^	0.97	2.59	3.476 (4)	152

Symmetry codes: (i) −*x*, −*y*, −*z*+1; (ii) *x*+1, *y*, *z*.

## References

[bb1] Akkurt, M., Çelik, Í., Cihan, G., Çapan, G. & Büyükgüngör, O. (2010). *Acta Cryst.* E**66**, o830. 10.1107/S1600536810009098 PMC2983761 21580658

[bb2] Akkurt, M., Karaca, S., Cihan, G., Çapan, G. & Büyükgüngör, O. (2009). *Acta Cryst.* E**65**, o1009–o1010. 10.1107/S1600536809012677 PMC2977697 21583833

[bb3] Bernstein, J., Davis, R. E., Shimoni, L. & Chang, N.-L. (1995). *Angew. Chem. Int. Ed. Engl.* **34**, 1555–1573.

[bb4] Cihan-Üstündağ, G. & Çapan, G. (2012). *Mol. Divers.* **16**, 525–539. 10.1007/s11030-012-9385-y 22893206

[bb5] Cremer, D. & Pople, J. A. (1975). *J. Am. Chem. Soc.* **97**, 1354–1358.

[bb6] Farrugia, L. J. (2012). *J. Appl. Cryst.* **45**, 849–854.

[bb7] Güzel, O., Terzioğlu, N., Çapan, G. & Salman, A. (2006). *Arkivoc*, **xii**, 98–110.

[bb8] Kaynak, F. B., Öztürk, D., Özbey, S. & Çapan, G. (2005). *J. Mol. Struct.* **740**, 213–221.

[bb9] Sheldrick, G. M. (2008). *Acta Cryst.* A**64**, 112–122. 10.1107/S0108767307043930 18156677

[bb10] Stoe & Cie (2002). *X-AREA* and *X-RED32* Stoe & Cie, Darmstadt, Germany.

